# Long noncoding RNA *Mhrt* alleviates angiotensin II-induced cardiac hypertrophy phenotypes by mediating the miR-765/Wnt family member 7B pathway

**DOI:** 10.1515/med-2023-0681

**Published:** 2023-05-12

**Authors:** Manli Yuan, Huaping Jia, Bei Zhao, Can Zhang, Xiaowen Zuo

**Affiliations:** Department of Ultrasound Medicine, Strategic Support Force Medical Center, Beijing, 100101, China; Department of Cardiovascular Medicine, Strategic Support Force Medical Center, Beijing, China

**Keywords:** cardiac hypertrophy, *Mhrt*, Ang II, miR-765, WNT7B

## Abstract

Long noncoding RNAs (lncRNAs) are known to participate in the pathological process of cardiac hypertrophy. This study aimed to investigate the function of the lncRNA, myosin heavy-chain associated RNA transcript (*Mhrt*), in cardiac hypertrophy and its possible mechanism of action. Adult mouse cardiomyocytes were treated with angiotensin II (Ang II) and transfected with *Mhrt*; cardiac hypertrophy was evaluated by estimating atrial natriuretic peptide, brain natriuretic peptide, and beta-myosin heavy-chain levels, and cell surface area by reverse transcription-quantitative polymerase chain reaction, western blotting, and immunofluorescence staining. The interaction between the *Mhrt*/Wnt family member 7B (WNT7B) and miR-765 was assessed using a luciferase reporter assay. Rescue experiments were performed by analyzing the role of the miR-765/WNT7B pathway underlying the function of *Mhrt*. The results indicated that Ang II induced hypertrophy of cardiomyocytes; however, overexpression of *Mhrt* alleviated the Ang II-induced cardiac hypertrophy. *Mhrt* acted as a sponge for miR-765 to regulate the expression of WNT7B. Rescue experiments revealed that the inhibitory effect of *Mhrt* on myocardial hypertrophy was abolished by miR-765. Additionally, the knockdown of WNT7B reversed the suppression of myocardial hypertrophy induced by downregulating miR-765. Taken together, *Mhrt* alleviated cardiac hypertrophy by targeting the miR-765/WNT7B axis.

## 1 Introduction

In an adult heart, cardiac hypertrophy is characterized by increased sizes of individual cardiomyocytes, which reduces ventricular wall pressure and maintains the normal function and efficiency of the heart [1,2]. In addition to physiological hypertrophy, pathological cardiac hypertrophy is associated with cardiac dysfunction, increased interstitial fibrosis, and cell death, which usually involves heart failure and sudden death [3,4]. As the density of capillaries decreases, blood supply is insufficient, leading to the transformation of physiological cardiac hypertrophy to pathological hypertrophy [5]. In recent years, many mediators involved in the processes of cardiac hypertrophy have been reported, such as the G protein, renin–angiotensin system, and the PI3K/AKT, MAPK, and NF-kappaB pathways [6]; however, the regulatory mechanisms remain unclear. As gene therapy is a novel therapeutic approach [7], it is necessary to explore more effective targets for the treatment of cardiac hypertrophy.

Long noncoding RNAs (lncRNAs) are noncoding RNA transcripts that are longer than 200 nucleotides. Increasing evidence suggests that dysregulation of lncRNAs is linked to cardiac hypertrophy, especially physiological hypertrophy [8,9]. In the pathological process, aberrant expression of lncRNAs is associated with sarcomere formation, calcium processing, and mitochondrial dysfunction, leading to genetic mutations, stress overload, inflammation, and endocrine abnormalities [10]. Additionally, lncRNAs are associated with cardiac remodeling [11]. Myosin heavy-chain associated RNA transcript (*Mhrt*), also named Myheart, is a transcript located on the Myh7 gene. *Mhrt* is cardiac-specific, found only in cardiomyocytes, and is present at low levels in fetal hearts but is abundantly present in adult hearts [12]. The level of *Mhrt* is elevated in cardiac myocytes of hearts that have undergone acute myocardial infarction and protects cardiomyocytes from apoptosis [13]. Low expression of *Mhrt* indicates a poor survival rate in patients with chronic heart failure [14]. In cardiac hypertrophy, *Mhrt* can inhibit myocardin expression and attenuate disease progression [15]. However, the regulatory roles of *Mhrt* in cardiac hypertrophy remain unclear, and further research is needed.

In this study, we aimed to explore the functions of *Mhrt* in the regulatory mechanisms underlying cardiac hypertrophy. We hypothesized that overexpression of *Mhrt* attenuated angiotensin II (Ang II)-induced cardiac hypertrophy. Additionally, *Mhrt* could bind to the microRNA miR-765 to regulate the expression of the Wnt family member 7B (WNT7B). Furthermore, the function of the downstream regulatory pathway miR-765/WNT7B was explored. The goal of the study provided a potential option, mediating the *Mhrt*/miR-765/WNT7B axis, to treat pathological cardiac hypertrophy.

## 2 Materials and methods

### 2.1 Bioinformatics analysis

The starbase online database (https://starbase.sysu.edu.cn/) was used to select the target miRNAs of *Mhrt* and the binding sites between miR-765 and the 3′-UTR of *Mhrt.* The TargetScan (https://www.targetscan.org/vert_80/) and miRDB (https://mirdb.org/) databases were used to obtain the genes targeted by miR-756 and the binding sites between miR-765 and the WNT7B. The Kyoto Encyclopedia of Genes and Genomes (KEGG) pathway analyses were used to identify the significant pathways of target genes.

### 2.2 Animal model

The animal experimental protocol was approved by the Ethics Committee of the Strategic Support Force Medical Center. Male C57BL/6 mice (6‒8 weeks old, 20‒22 g) were divided into control and Ang II groups (six mice per group). The mice in the Ang II group were administered 1.46 mg/kg/day Ang II (Sigma**-**Aldrich, St. Louis, MO, USA) for 2 weeks by implanting osmotic mini-pumps in the peritoneal cavity. Similarly, mice in the control group were administered saline for 2 weeks. All the mice were sacrificed by cervical dislocation, and their hearts were removed and weighed after washing with PBS.

### 2.3 Hematoxylin–eosin (H&E) staining assay

H&E staining was performed as described previously [16]. Isolated hearts were fixed with 4% paraformaldehyde for 24 h. Then, the cardiac tissues were cut into 5 μm sections after paraffin embedding. All sections were stained with hematoxylin for 10 min and eosin for 1 min. The stained sections were visualized under a microscope.

### 2.4 Cardiomyocyte culture and Ang II treatment

Adult mouse cardiomyocytes obtained from SUNNCELL (Wuhan, China, Catalog No. SNP-M013) were incubated in a cardiomyocyte complete culture medium (Catalog No. SNPM-M013; SUNNCELL) at 37°C with 5% CO_2_. Next, to induce hypertrophy, after the cells were cultured in a serum-free medium for 24 h, different concentrations of Ang II (10^−5^, 10^−6^, 10^−7^, and 10^−8^ mol/l) were incubated with the myocardial cells at 37°C for 48 h.

### 2.5 Cell transfection

siRNA-negative control (si-nc: 5′-UAUCGCCGUAGACCCACU-3′), si-Mhrt (5′-GAGUGUGCACAAGAGAAAU-3′), vector, *Mhrt* vector, mimic nc (5′-UUAUCUCCUGUGCGATT-3′), inhibitor nc (5′-CAGUACAUUGGUUCUGCAA-3′), miR-765 mimic (5′-UGGAGGAGAAGGAAGGUGAUG-3′), miR-765 inhibitor (5′-CAUCACCUUCCUUCUCCUCCA-3′), and si-WNT7B (5′-TGTTTCTCTGCTTTGGCGTCCTGTA-3′) were transfected into cells using the Lipofectamine 2000 Transfection Reagent (Catalog No. 11668-019; Invitrogen, Carlsbad, CA, USA). At 48 h post-transfection, the cell transfection efficiency was measured by reverse transcription-quantitative polymerase chain reaction (RT-qPCR).

### 2.6 RT-qPCR

TRIzol reagent (Catalog No. 15596026; Invitrogen) was used for the isolation of total RNA. Reverse transcription of miRNA was performed using the All-in-One^TM^ miRNA First-Strand cDNA Synthesis Kit (Catalog No. QP113), and reverse transcription of *Mhrt* and mRNA was conducted using the SureScripts™ First-Strand cDNA Synthesis Kit (Catalog No. QP057). Subsequently, qPCR was performed using All-in-One™ miRNA qRT-PCR Detection Kit (Catalog No. QP116) or All-in-One™ qPCR Mix (Catalog No. QP002). The kits used in this study were purchased from GeneCopoeia (Guangzhou, China). The relative expression was calculated using the 2^−∆∆CT^ method. U6 was used as an internal control for miR-765, while GAPDH was used as a housekeeping gene for *Mhrt* and other mRNAs.

### 2.7 Western blotting

All the kits or reagents for western blotting were obtained from Elabscience (Wuhan, China). Cells were lysed using the RIPA lysis buffer, and the protein concentration was estimated using the BCA Protein Concentration Detection Kit (Catalog No. E-BC-K318). SDS-PAGE (10%; Catalog No. E-IR-R305) was employed to separate proteins, which were then transferred onto PVDF membranes. The membranes were blocked for 1.5 h in 5% skim milk. The membranes were then incubated with primary antibodies (anti-ANP: ab209232, 1:1,000; anti-BNP: ab236101, 1:2,000; anti-β-MHC: ab180779, 1:2,000; and anti-β-actin: ab8227, 1:5,000; Abcam, Cambridge, CA, USA) overnight at 4°C and then incubated with secondary antibodies (Catalog No. E-AB-1003; Elabscience) for 1 h at 25°C. Protein bands were visualized using an ECL luminous detection solution (Catalog No. E-BC-R347). Gray analysis was performed by normalizing to GAPDH levels.

### 2.8 Immunofluorescence (IF) staining

IF staining was performed as described previously [16]. After 48 h of transfection, the cells were fixed with 4% formaldehyde and permeabilized in 0.5% Triton X-100 for 20 min at room temperature. The cells were then washed with PBS and blocked with normal goat serum for 30 min. Primary antibody (α-actinin: ab90421, 1:100; Abcam) was added to the cells, and the cells were incubated overnight at 4°C. The next day, the cells were incubated with a secondary antibody (Goat Anti-Mouse IgG H&L [Alexa Fluor^®^ 594]: ab150116, 1:500; Abcam) at room temperature for 1 h. IF was detected using a fluorescence microscope (Olympus, Tokyo, Japan) after the cells were incubated with DAPI for 10 min. One hundred cells from three wells were randomly selected to quantify the surface area using Image-Pro Plus 6.0.

### 2.9 Luciferase reporter assay

The binding sites between Mhrt and miR-765 were predicted using the Starbase database, and the binding sites between miR-765 and WNT7B were predicted using the TargetScan database. HEK293T cells were seeded into 24-well plates. *Mhrt*-wild type (WT) and WNT7B-WT containing the predicted binding sites of miR-765 were inserted into pGL3 vectors, and their corresponding mutant (MUT) sequences were also cloned into pGL3 vectors. These recombinant plasmids were co-transfected with the miR-765 mimic and mimic NC into HEK293T cells using Lipofectamine 2000 Transfection Reagent (Invitrogen). After 48 h, relative luciferase activity was analyzed using the Dual-Luciferase Reporter Assay System (Catalog No. E1960; Promega, Madison, WI, USA).

### 2.10 Statistical analysis

All experiments were repeated at least three times, and the data are represented as mean ± SD. GraphPad Prism 6.0 software (GraphPad, CA, USA) was used for the analysis. Significant differences were determined using the Student’s *t*-test (between two groups) and one-way ANOVA (between multiple groups). *P* < 0.05 was considered to be statistically significant.

## 3 Results

### 3.1 *Mhrt* was decreased in Ang II-induced hypertrophy of cardiomyocytes

**Figure 1:**
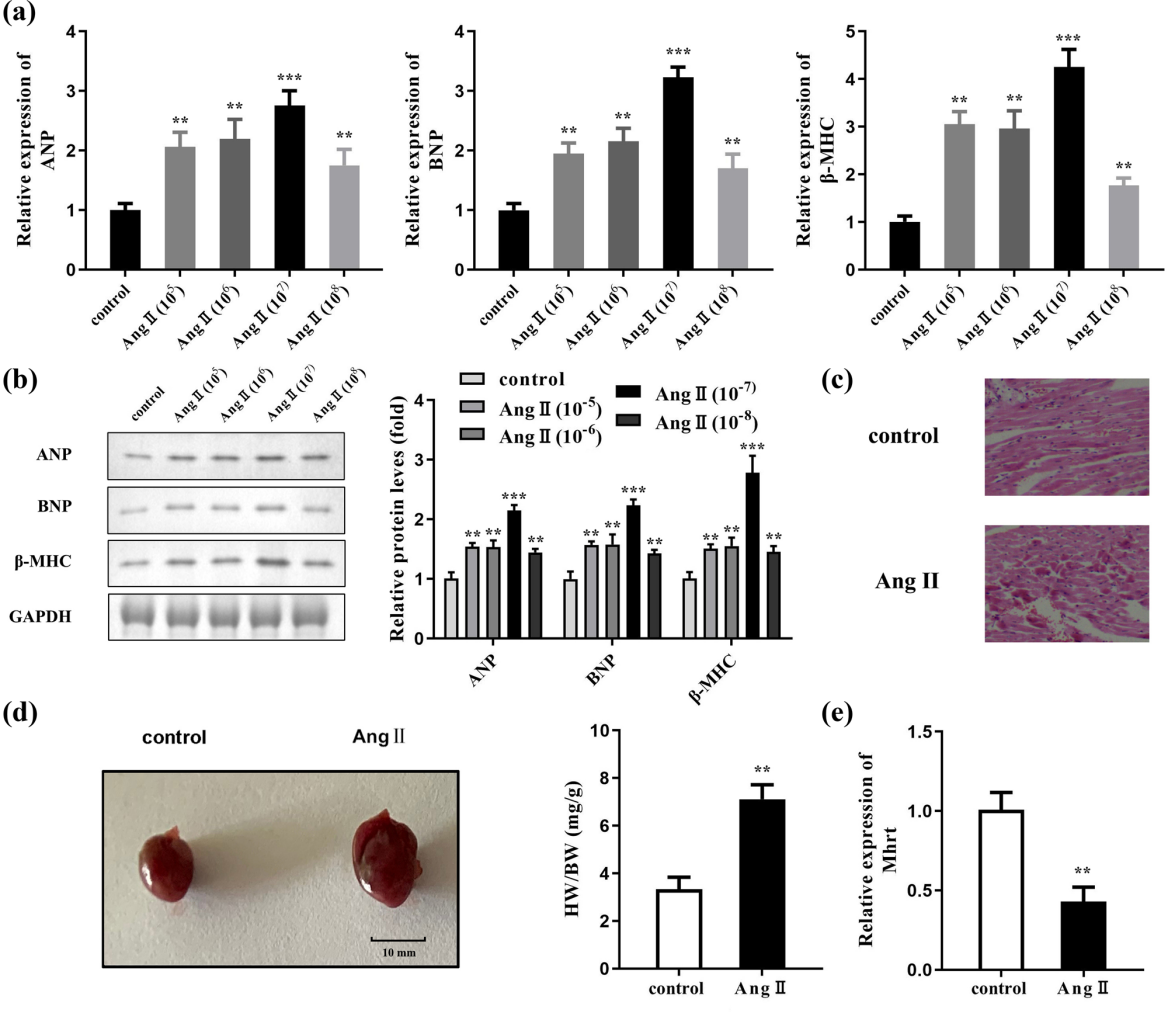
*Mhrt* was elevated in Ang II-induced cardiac hypertrophy. (a) RT-qPCR was carried out for the detection of ANP, BNP, and β-MHC expression. (b) ANP, BNP, and β-MHC levels were assessed by western blotting, and their levels were quantified. (c) The size of cardiomyocytes was analyzed using H&E staining assay. (d) Images of the hearts obtained from mice in the control and Ang II-treated groups, and the ratio of heart weight to body weight (HW/BW) was quantified. (e) *Mhrt* expression was analyzed by RT-qPCR in cardiomyocytes treated with Ang II. ***P < 0.001; **P < 0.01.

First, cardiomyocytes were exposed to different concentrations of Ang II (10^−5^, 10^−6^, 10^−7^, and 10^−8^ mol/l) for 48 h, and cardiomyocyte hypertrophy was evaluated. The results of RT-qPCR showed that the mRNA expression levels of atrial natriuretic peptide (ANP), brain natriuretic peptide (BNP), and beta-myosin heavy chain (β-MHC) in the Ang II-treated group were significantly increased than those in the control group (Figure 1a). Similarly, the protein levels of ANP, BNP, and β-MHC were also significantly higher in Ang II-treated cells (Figure 1b). The most significant Ang II concentration (10^−7^ mol/l) was used in subsequent cell experiments. The results of the H&E staining assay showed that the size of the cardiomyocyte was increased (Figure 1c). In addition, Ang II treatment caused the heart to grow bigger, which in turn increased the ratio of heart weight/body weight (Figure 1d). Moreover, the expression of *Mhrt* was reduced in cardiomyocytes treated with Ang II, as analyzed by RT-qPCR (Figure 1e).

### 3.2 Overexpressed *Mhrt* alleviated Ang II-induced cardiac hypertrophy

**Figure 2:**
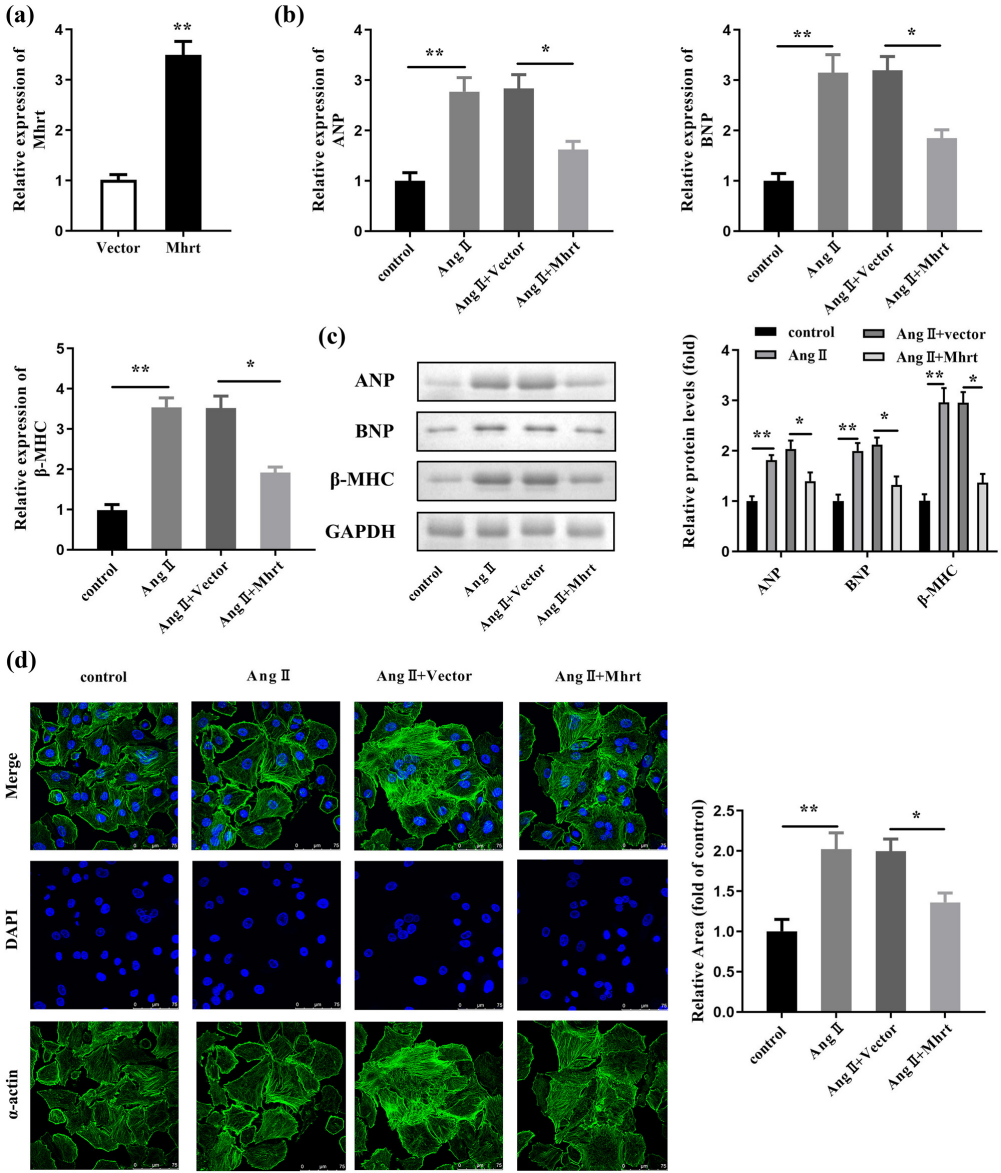
*Mhrt* attenuated cardiac hypertrophy induced by Ang II. (a) Transfection efficiency was tested by RT-qPCR after transfection with *Mhrt* overexpressing vector. (b) ANP, BNP, and β-MHC mRNA expression levels were evaluated using RT-qPCR. (c) ANP, BNP, and β-MHC levels were evaluated using western blotting and quantified with GAPDH as an internal control. (d) Cell surface area was visualized using IF staining and quantified. **P < 0.01; *P < 0.05.

To explore the functions of *Mhrt*, cardiac myocytes were transfected with the *Mhrt* vector and treated with Ang II. The RT-qPCR data indicated that *Mhrt* was significantly upregulated after transfection of the *Mhrt* overexpression vector (Figure 2a). Meanwhile, ANP, BNP, and β-MHC were elevated by Ang II both at the mRNA and protein levels, which were rescued by overexpressed *Mhrt* (Figure 2b and c). Ang II remarkably increased the cell surface area, which was significantly alleviated by overexpressed *Mhrt* (Figure 2d).

### 3.3 miR-765 functioned as the target of *Mhrt*

**Figure 3:**
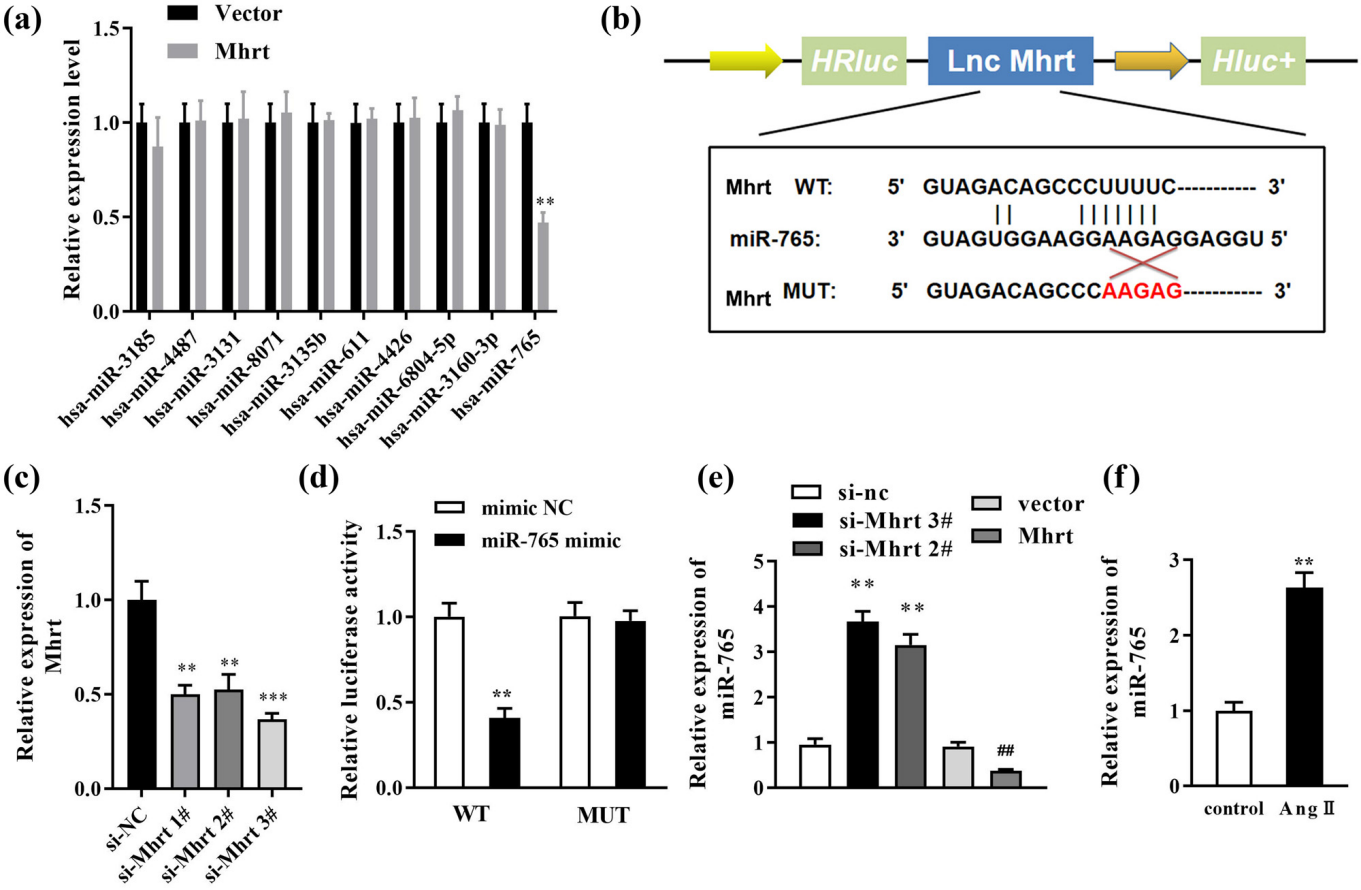
Targeting effect of *Mhrt* to miR-765. (a) The levels of top-10 target miRNAs of *Mhrt* were detected by RT-qPCR after *Mhrt* overexpression. (b) Complementary sequences of *Mhrt* and miR-765 are shown in accordance with bioinformatics analysis. (c) Verification of transfection efficiency of si-*Mhrt* 1#, 2#, and 3#. (d) Verification of targeting relationship using luciferase reporter assay. (e) miR-765 level was assessed using RT-qPCR in cells transfected by si-*Mhrt* or *Mhrt* vector. (f) miR-765 expression was analyzed by RT-qPCR in cardiomyocytes treated with Ang II. **P < 0.01. ^##^ *P* < 0.01.

Through the starbase database (https://starbase.sysu.edu.cn/), we obtained the top-10 target miRNAs of *Mhrt*, and after *Mhrt* overexpression, just the miR-765 expressions were decreased while other miRNAs showed no difference (Figure 3a). Besides, the binding sites between miR-765 and the 3′-UTR of *Mhrt* were obtained from the starbase database, and *Mhrt*-MUT sequences were designed (Figure 3b).In addition, the transfection efficiency of si-*Mhrt* was tested, and we found the *Mhrt* levels were significantly decreased after si-*Mhrt* transfection. Si-*Mhrt* 3# was selected for the next experiments because of the best transfection efficiency (Figure 3c). Luciferase reporter assay demonstrated that co-transfection with the miR-765 mimic and *Mhrt* 3′-UTR WT significantly reduced luciferase activity, while in the *Mhrt*-MUT group, a significant change in the luciferase activity was not observed (Figure 3d). Silencing *Mhrt* (si-*Mhrt* 2# and si-*Mhrt* 3# transfection) significantly increased the expression of miR-765 but the overexpression of *Mhrt* remarkably reduced miR-765 expression (Figure 3e). In Ang II-treated cells, the miR-765 expression was significantly higher than that in the control group (Figure 3f).

### 3.4 Inhibitory effect of *Mhrt* on cardiac hypertrophy was abolished by miR-765 in cardiomyocytes

**Figure 4:**
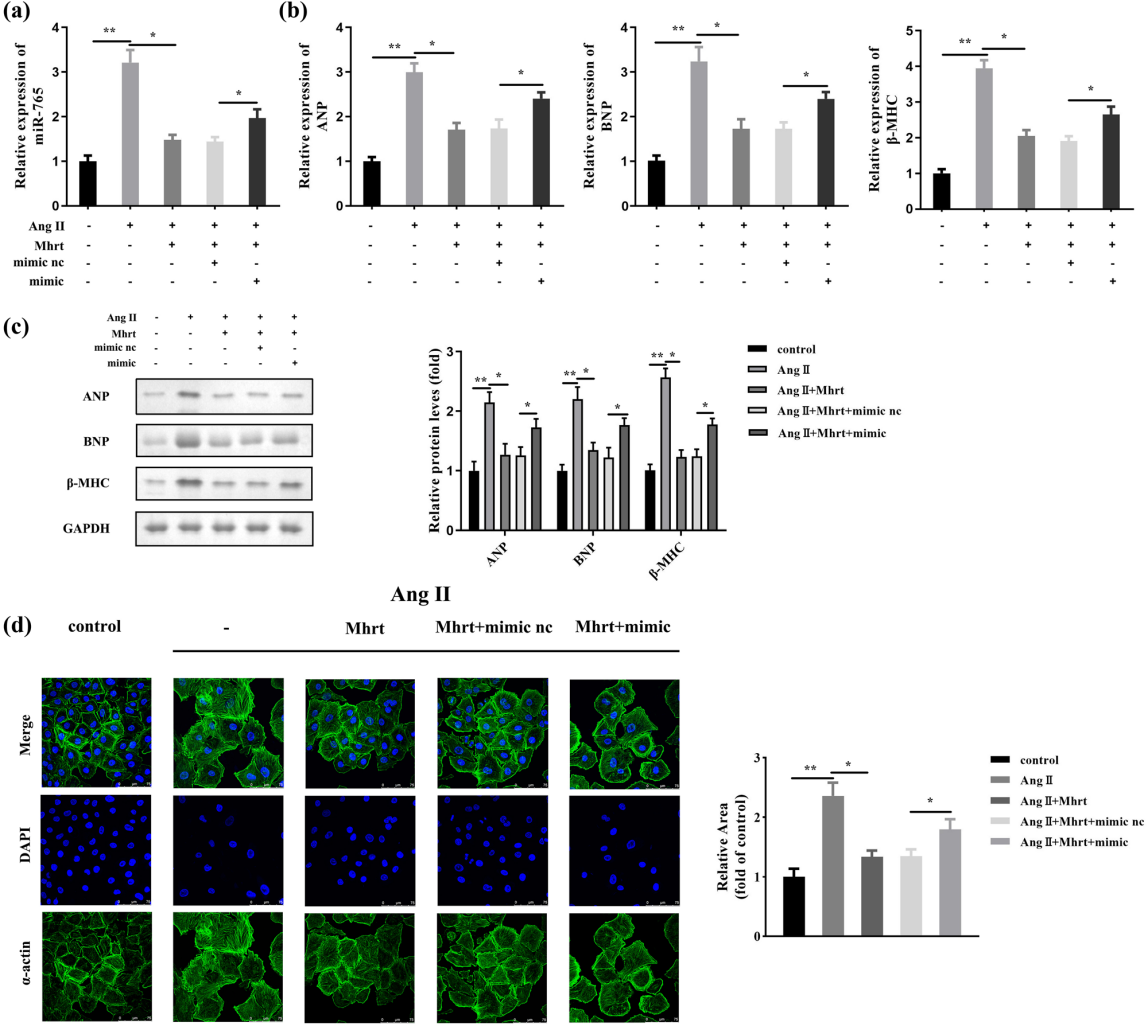
*Mhrt* attenuated cardiac hypertrophy through sponging of miR-765. (a) Transfection efficiency was assessed by RT-qPCR. (b) Relative mRNA expression of ANP, BNP, and β-MHC was assessed by RT-qPCR. (c) Relative protein levels of ANP, BNP, and β-MHC were assessed using western blotting and quantified. (d) Cell surface area was visualized using IF staining and quantified. **P < 0.01; *P < 0.05.

In transfected cells, Ang II increased the miR-765 level, which was suppressed by *Mhrt*; however, this suppression was abrogated by miR-765 mimic (Figure 4a). Furthermore, overexpression of miR-765 partially abolished the inhibition of ANP, BNP, and β-MHC by *Mhrt* in Ang II-induced hypertrophic cells (Figure 4b and c). The cardiomyocyte phenotype was altered by *Mhrt*, which was rescued by the miR-765 mimic in Ang II-induced myocardial hypertrophy (Figure 4d).

### 3.5 miR-765 directly targeted WNT7B

**Figure 5:**
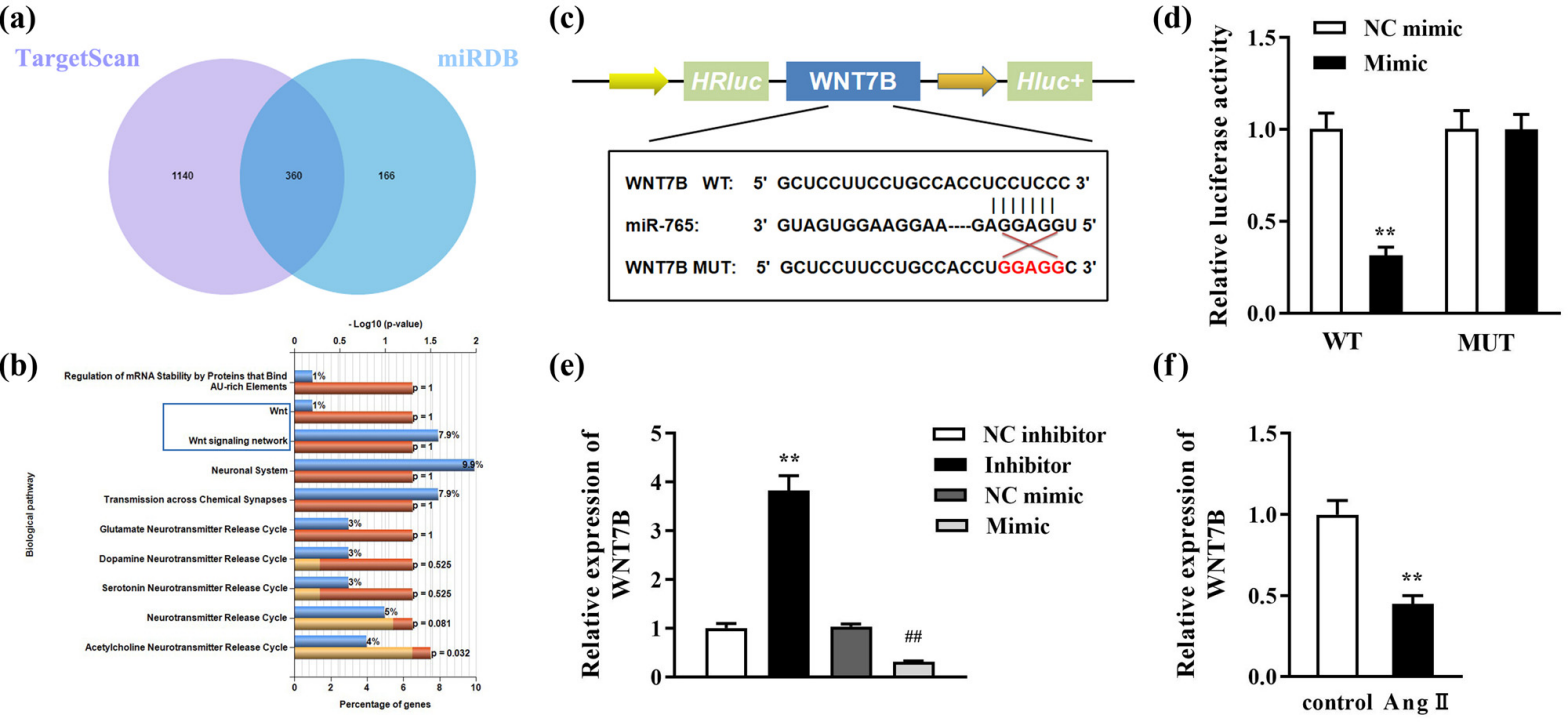
WNT7B is negatively correlated with miR-765. (a) Venn diagram of target genes in the TargetScan and miRDB database. (b) KEGG pathway analysis. (c) The possible binding sites between miR-765 and WNT7B were predicted through bioinformatics analysis. (d) The targeted relationship was verified using luciferase reporter analysis. (e) MiR-765 inhibitor was used to inhibit the miR-765 expression, and miR-765 mimic was used to increase miR-765 expression; the WNT7B level was measured in miR-765-overexpressing or miR-765-nonexpressing cells. (f) WNT7B level was detected in cells treated with Ang II using RT-qPCR. **P < 0.01; ^##^ *P* < 0.01.

Through the TargetScan (https://www.targetscan.org/vert_80/) and miRDB (https://mirdb.org/) databases, we obtained 360 genes targeted by miR-756 (Figure 5a). Then, through the KEGG pathway analysis, we found that WNT7B was enriched in the Wnt signaling pathway, which was selected for the next experiments (Figure 5b). The possible binding sites between miR-765 and WNT7B were obtained, and WNT7B-MUT sequences were designed (Figure 5c). Additionally, cells co-transfected with the miR-765 mimic and WNT7B-WT displayed significantly decreased luciferase activity (Figure 5d). Then, the miR-765 inhibitor was used to inhibit miR-765 expression, and we found that the miR-765 inhibitor significantly upregulated WNT7B levels, whereas the miR-765 mimic notably downregulated WNT7B (Figure 5e). Furthermore, the expression of WNT7B was significantly decreased in Ang II-treated cells (Figure 5f).

### 3.6 Knockdown of WNT7B reversed the effects of miR-765 on cardiac hypertrophy

**Figure 6:**
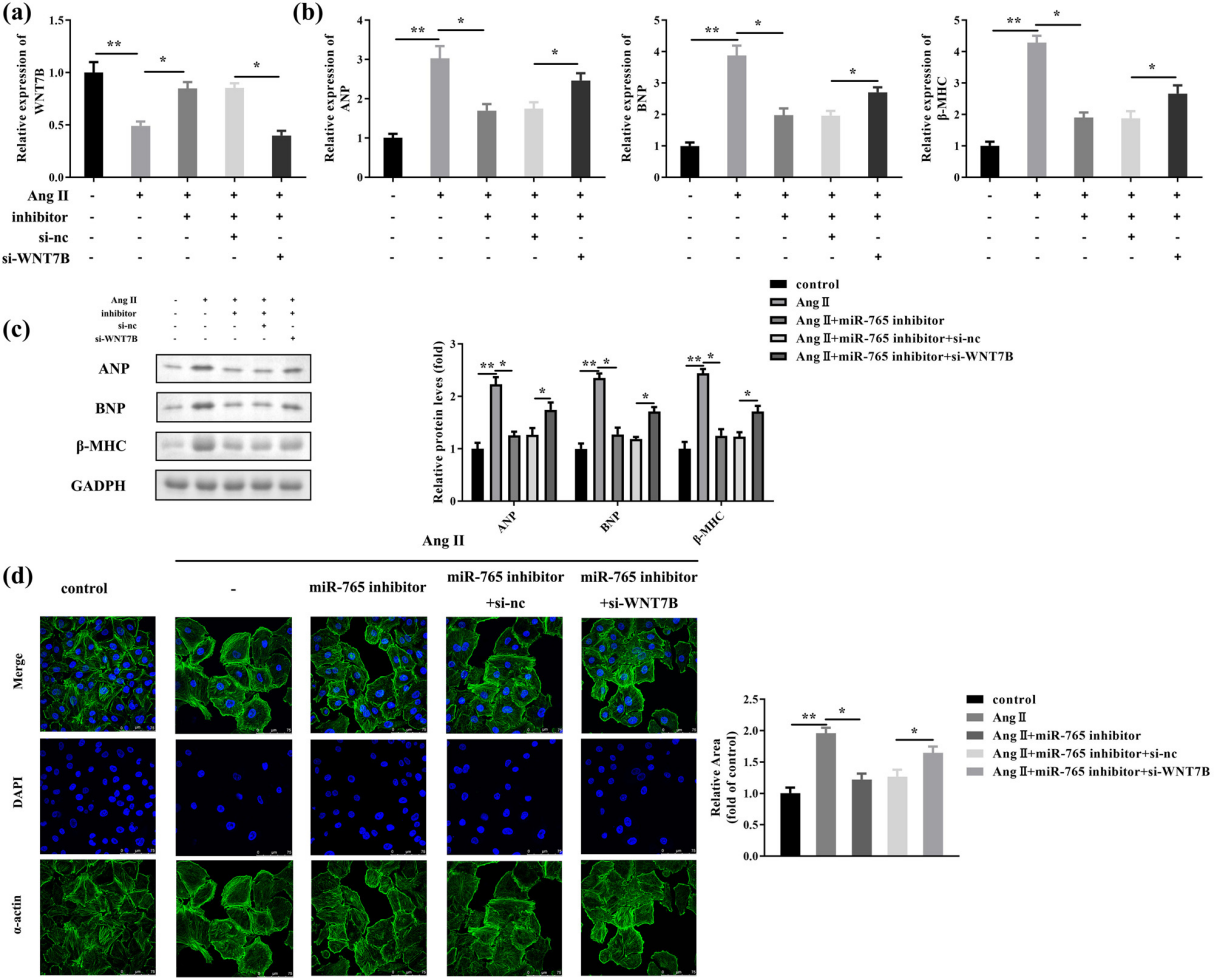
Downregulation of miR-765 alleviated cardiac hypertrophy via the targeting of WNT7B. (a) Transfection efficiency was analyzed using RT-qPCR. (b) The mRNA expression levels of ANP, BNP, and β-MHC were evaluated using RT-qPCR. (c) The protein levels of ANP, BNP, and β-MHC were quantified using western blotting. (d) Cell surface area was assessed using IF staining. **P < 0.01; *P < 0.05.

**Figure A1:**
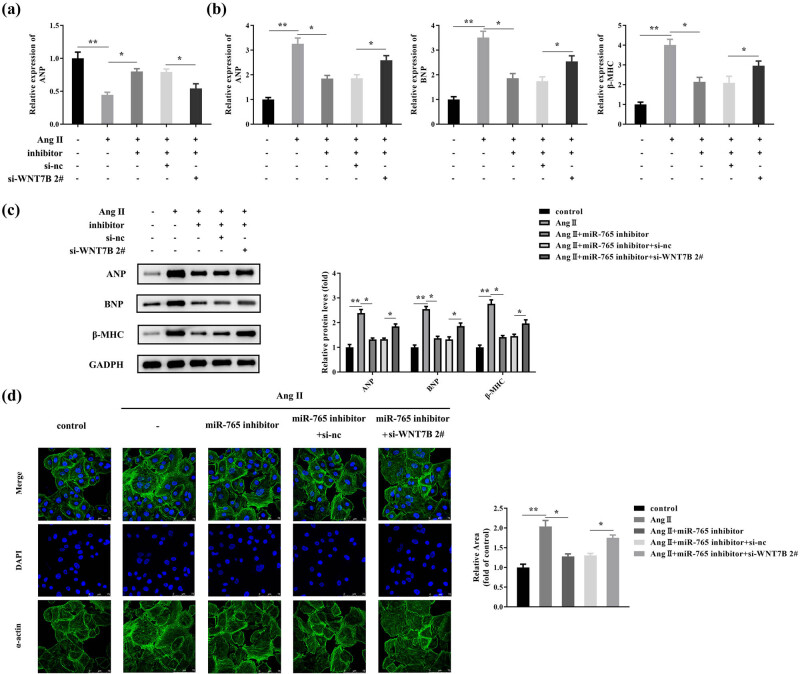
Downregulation of miR-765 alleviated cardiac hypertrophy via the targeting of WNT7B. (a) Transfection efficiency was analyzed using RT-qPCR. (b) The mRNA expression levels of ANP, BNP, and β-MHC were evaluated using RT-qPCR. (c) The protein levels of ANP, BNP and β-MHC were quantified using western blotting. (d) Cell surface area was assessed using IF staining. **P < 0.01. *P < 0.05.

**Figure A2:**
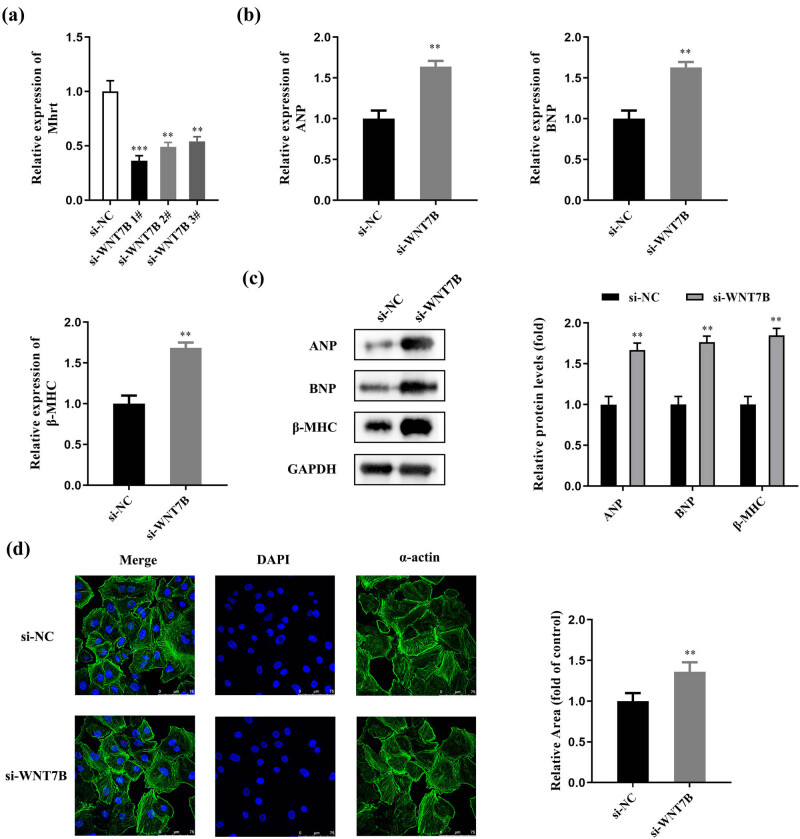
WNT7B knockdown induced hypertrophy of cardiomyocytes. (a) Transfection efficiency of si-WNT7B was analyzed using RT-qPCR. (b) ANP, BNP and β-MHC mRNA expression levels were evaluated using RT-qPCR. (c) ANP, BNP, and β-MHC levels were evaluated using western blotting and quantified with GAPDH as an internal control. (d) Cell surface area was visualized using IF staining and quantified. **P < 0.01, ***P < 0.001.

After transfection, WNT7B expression was decreased by Ang II but was increased by miR-765 inhibitor; this increase was subsequently abolished by si-WNT7B (Figure 6a). The levels of ANP, BNP, and β-MHC were repressed by the downregulation of miR-765 in Ang II-induced hypertrophic cells; this effect was abrogated by silencing WNT7B (Figure 6b and c). In addition, the inhibitory effect on the cardiomyocyte size induced by the miR-765 inhibitor was reversed by si-WNT7B (Figure 6d). Additionally, we also performed these experiments using another si-WNT7B 2# and obtained the same results (Figure A1). Moreover, we found that WNT7B knockdown, like ANG treatment, also induced hypertrophy of cardiomyocytes (Figure A2).

## 4 Discussion

Increasing evidence has demonstrated that lncRNAs are associated with cardiovascular diseases, including cardiac hypertrophy [8,9]. In the present study, we explored the function of *Mhrt* in cardiac hypertrophy and found that *Mhrt* could alleviate Ang II-induced cardiac hypertrophy via the miR-765/WNT7B pathway.

The pathological cardiac hypertrophy process is usually accompanied by the release of ANP, BNP, and β-MHC, as well as enlargement of the surface area of cardiomyocytes [17,18]. In this study, we found that Ang II enhanced ANP, BNP, and β-MHC, and also increased the cell surface area, suggesting that Ang II induced hypertrophy of cardiomyocytes.

Dysregulation of Mhrt is associated with the pathogenesis of cardiomyopathy, such as cardiac hypertrophy, heart failure, and cardiac fibrosis [19,20]. Mhrt is highly enriched in the nucleus of cardiomyocytes and downregulated by pressure overload. Inhibition of Mhrt transcription and restoring Mhrt levels to pre-stress levels protect the heart from hypertrophy and failure [12]. Previous studies have shown that different mechanisms of *Mhrt* are involved in cardiac hypertrophy. For example, maintaining *Mhrt* at pre-stress levels protects the heart from hypertrophy by inhibiting the activation of the Brg1-Hdac-Parp chromatin repressor complex [12]. Besides, an interaction between *Mhrt* and myocardin could suppress cardiac hypertrophy, including the effect of *Mhrt* on the acetylation of myocardin and myocardin-activating *Mhrt* transcription [21]. Unlike the findings mentioned above, Xu et al. [15] suggested that *Mhrt* represses myocardin expression by regulating the miR-145a-5p/KLF4 pathway, leading to myocardial hypertrophy. Additionally, several lncRNAs also regulate cardiac hypertrophy, such as lncRNA AK006774 and NEAT1 [22,23]. Based on these previous studies, we investigated the function of *Mhrt* in cardiac hypertrophy by estimating ANP, BNP, and β-MHC levels, and the cell surface area. We found that *Mhrt* levels decreased in Ang II-induced cardiac hypertrophy. Overexpressed *Mhrt* inhibited ANP, BNP, and β-MHC levels, as well as cell surface area in Ang II-treated myocardial cells, suggesting that *Mhrt* could alleviate Ang II-induced cardiac hypertrophy.

MiR-765 is a microRNA located on chromosome 1. Many previous studies have examined the role of miR-765 in numerous diseases. For instance, in malignancy, miR-765 commonly functions as a tumor suppressor or tumor promoter and acts as a prognosis biomarker [24–26]. miR-765 inhibited the protective effect of cerebral ischemia/reperfusion injury induced by the knockdown of lncRNA FOXD3-AS1 [27]. Additionally, miR-765 is involved in cardiovascular diseases; it is found to be highly expressed in patients with coronary heart disease [28]. In geriatric coronary artery disease, miR-765 levels are observed to be upregulated; these values are important for the clinical diagnosis of this disease [29]. Moreover, high expression of miR-765 is conducive to heart failure [30]. In the present study, miR-765 was upregulated in Ang II-treated myocardial cells; however, *Mhrt* was able to sponge miR-765. Functionally, miR-765 abolishes the inhibitory effect of *Mhrt* on ANP, BNP, and β-MHC levels and induces enlargement of the cardiomyocyte surface area, suggesting that *Mhrt* attenuated Ang II-induced cardiac hypertrophy by sponging miR-765 in this study.

Wnt signaling is usually activated during cardiac development, cardiac hypertrophy, myocardial infarction, and heart failure [31]. WNT7B, a member of the Wnt family, encodes a Wnt signaling ligand. WNT7B has been reported to promote choroidal neovascularization, to be involved in vascular calcification, and is associated with pulmonary arteriolar remodeling induced by hypoxia [32–34]. However, the role of WNT7B in cardiac hypertrophy remains unknown. In the current study, WNT7B was identified to be a target of miR-765, and its level was downregulated in Ang II-induced cardiac hypertrophy. Furthermore, the knockdown of WNT7B abrogated the inhibitory effect on ANP, BNP, and β-MHC levels and cell surface area induced by the downregulation of miR-765. Taken together, the downregulation of miR-765 targeted WNT7B to suppress Ang II-induced cardiac hypertrophy.

## 5 Conclusion

LncRNA *Mhrt* is downregulated in cardiac hypertrophy. Overexpression of *Mhrt* protected against Ang II-induced cardiac hypertrophy via the miR-765/WNT7B axis. These findings suggest that mediation of the *Mhrt*/miR-765/WNT7B axis can be used as a treatment strategy for pathological cardiac hypertrophy.
